# Authority, favoritism, and fairness: how paternalistic leadership and nepotism shape nurses’ perceptions of justice

**DOI:** 10.1186/s12912-025-03888-y

**Published:** 2025-10-30

**Authors:** Hend Sabry Saad Habaza, Heba Rabea Hagras, Hanan Elsaid Elsabahy, Manal Saleh Moustafa Saleh, Hamad Ghaleb Dailah, Ahmed Hendy, Hussain Ali Shobaili

**Affiliations:** 1 Samanoud Central Hospital, Mansoura, Egypt; 2 Nursing Administration, Faculty of Nursing, Mansoura University, Mansoura, Egypt; 3 Nursing Administration, Faculty of Nursing, Zagazig University, Zagazig, Egypt; 4 College of Nursing and Health Sciences, Jazan University, Jazan, Saudi Arabia; 5 Department of Computational Mathematics and Computer Science, Institute of Natural Sciences and Mathematics, Ural Federal University, Yekaterinburg, Russian Federation; 6 Department of Mechanics and Mathematics, Western Caspian University, Baku, 1001 Azerbaijan

**Keywords:** Paternalistic leadership, Nepotism, Perception of justice, Staff nurses

## Abstract

**Background:**

Paternalistic leaders serve as role models for their followers so that they understand the kind of behavior that is ethically unacceptable as nepotism and understand how ethical issues are handled as organizational justice.

**Aim:**

This study aimed to investigate the effects of Paternalistic Leadership and Nepotism on Nurses’ Perception of Justice.

**Method:**

A descriptive correlational design was employed with a Convenience sample of 267 staff nurses from one hospital, who had a minimum of two years of professional experience in the field and were willing to participate in the study. This prerequisite helps ensure that participants have a deep, practical understanding of the hospital’s culture and management. A nurse with two years of experience has moved beyond the initial training and orientation phase. They have a more comprehensive view of the workplace dynamics and have likely witnessed or been affected by various personnel decisions. Three instrument tools were used for data collection: The Paternalistic Leadership Questionnaire, which consisted of 15 items representing three dimensions; the Nepotism Questionnaire, which consisted of 12 items; and the Organizational Justice Questionnaire, which consisted of 44 items in five dimensions.

**Results:**

A statistically significant positive correlation was found between total paternalistic leadership style and organizational justice (*r* = 0.549), and a statistically significant negative correlation was found between total nepotism practice and organizational justice (*r* = -0.337). More than half of the studied nurses perceive a moderate level of paternalistic leadership, less than half perceive a moderate level of nepotism practice, and less than half perceive a moderate level of organizational justice.

**Conclusion:**

Based on the results of this study, we can conclude that the characteristics of paternalistic leadership, in the form of a good personality, being responsible, disciplined, and unselfish, as well as providing an example for subordinates. These moral values of paternalistic leaders regulate the ethical approach to decision-making and can create a fair environment, and be free from nepotism in an organization.

**Recommendations:**

Develop a comprehensive onboarding program that includes orientation sessions, job-specific training, and mentorship programs related to paternalistic leadership style, nepotism practices, and principles of organizational justice.

**Implications for nursing:**

Fairness is a critical leadership tool. Proactive nursing management must address issues of perceived injustice and nepotism head-on to build a resilient, engaged, and high-performing nursing staff. When nurses feel that leadership decisions are unfair or that nepotism is at play, it erodes morale and fuels burnout.

**Clinical trial number:**

Not applicable.

## Introduction

Since nursing is the foundation of patient care, effective leadership is essential to enhancing the caliber and sustainability of healthcare services [1, 2]. According to research, nurses’ job happiness and performance are significantly impacted by leadership styles, which in turn affects the standard of patient care [3]. Nonetheless, in many healthcare systems, leadership issues such as nepotism and unfit appointments continue to be major obstacles that impair organizational effectiveness and service quality [4].

The nursing profession in Egypt suffers from a number of difficulties, such as a lack of qualified personnel, insufficient rewards, and high rates of burnout. According to local research, leadership styles in Egyptian healthcare facilities have a significant impact on how well nurses work. For example [5], discovered that whereas authoritarian leadership is linked to higher job stress and lower productivity, participative leadership improves nurse satisfaction and performance. In a similar vein [6], stressed the significance of strong leadership in tackling the structural issues that Egyptian nursing staff face.

The difficulties in the nursing field are further exacerbated by reports that indicate nepotism plays a major role in the recruitment of leaders who lack the requisite administrative abilities [7]. These problems highlight how urgently more study is needed to examine how leadership philosophies and nepotism affect nurse performance in Egyptian hospitals. To improve healthcare results, this study attempts to pinpoint practical methods for creating a more encouraging and fair workplace.

Improving patient outcomes, job happiness, and nurse performance all depend on a positive and encouraging nursing work environment. According to research, a positive work environment that is marked by sufficient staffing, strong leadership, collaboration, resource accessibility, and recognition greatly lowers burnout, boosts retention, and encourages creativity among nurses [8, 9]. Additionally, by encouraging a culture of safety and teamwork, these settings help nurses grow both personally and professionally while delivering high-quality care.

When putting these changes into practice, cultural considerations also need to be taken into account. Interpersonal interactions and hierarchical structures are valued in Egypt’s high-context, collectivist culture, which could affect how leadership methods are viewed and implemented [10]. For example, even if participative leadership might work, it needs to be modified to accommodate cultural norms that value authority and deference to authority. In order to create culturally appropriate solutions, future studies should investigate how cultural factors affect the relationship between organizational justice, nepotism, and leadership styles.

Leadership is an essential managerial function due to its crucial role in the success of organizations. Paternalistic leadership (PL), one of the many varieties of leadership, is seen as an effective management strategy that attempts to foster a family-like environment at work. They establish a bond with their workers akin to that of a parent and son or brother and treat them like members of the family [11]. Paternalistic leadership is defined as a category of leadership that “combines strong discipline and authority with paternal benevolence” [12].

In other words, paternalist leaders are those who exert significant influence over their staff, allow them to voice their own opinions, permit collaborative decision-making, maintain control over them, and foster an inventive work environment [13]. Three key components make up paternalistic leadership: benevolence, moral leadership, and authoritarianism. Authoritarianism is the behavior of a leader who demands total obedience from their followers and exerts total authority over them [14]. When a leader exhibits benevolence, their actions show a personalized, all-encompassing concern for the welfare of their subordinates and their families. Kind leaders enable staff members to feel less stressed at work and have more energy for their jobs [15]. Because it prioritizes ethical integrity over all other considerations, ethical leadership is comparable to moral leadership. Leaders need to act ethically and provide an example for their followers to encourage adherence to their values and positive actions. Ethical principles, in this context, are characterized by equality, self-determination, and honesty [16, 17].

This study’s theoretical framework is based on well-established organizational justice theories, which offer a comprehensive lens through which to view the connection between employee attitudes, paternalistic leadership, and nepotism. The main theoretical pillar is the Social Exchange Theory [18], which holds that workers assess their interactions at work through a reciprocal exchange process in which their attitudes and actions are directly influenced by how fairly they view their treatment. By establishing unequal trade connections, nepotism undermines the fundamental idea of reciprocity and causes employees to perceive violations of distributive and procedural justice [19]. Additionally, employees compare their input-to-outcome ratios with those of others, especially family members of leaders who might receive preferential treatment, as explained by Equity Theory [19]. When familial ties impact organizational decisions, this comparison process becomes especially salient under paternalistic leadership, where leaders take on a fatherly position that can either strengthen emotions of unfairness or improve views of benign care [20]. The combination of these two frameworks offers a strong theoretical basis for investigating these intricate workplace dynamics by illuminating the ways in which paternalistic leadership behaviors and nepotistic practices combine to influence employees’ views of organizational justice.

There is an analogy in the relationship of follower and paternalistic leadership and father-child relation. But in terms of dealing with staff members, executives might not behave in the same way as parents do. It is forbidden for a leader to treat all of their followers equally while bestowing authority or kindness. In that instance, he or she is said to have given someone an unjust benefit on purpose. Consequently, any type of discrimination or nepotism at work is likely to be disguised as this fatherly leadership style in the event of any discriminatory treatment [11].

The definition of nepotism is the employment and promotion of unfit relatives based only on their relationship to a shareholder, official, or employer [21]. Thus, nepotism only emerges when one takes into account the relationship of affinity and kinship, independent of an individual’s abilities, skills, education level, or experience. Numerous areas, including hiring, promoting, paying employees, evaluating their performance, enforcing disciplinary actions, and using organizational resources, are subject to nepotism practices. In human resources, lower-level nepotism frequently occurs in hiring and assigning employees, rating their performance, and setting pay scales. Nepotism in human resources at the highest level manifests itself in senior manager elections, appointments, and promotions [22]. Nepotism lowers employees’ sense of equity toward their organization and demotivates them. Therefore, managers need to be very careful with nepotism to create a sense of organizational justice in the minds of employees [23].

An action or choice that is deemed morally correct based on principles of ethics, religion, justice, fairness, or the law is acknowledged as being just [24]. The word “justice” suggests that a behavior or activity is just or fair. The phrase “organizational justice” was initially used in organizational contexts and describes how workers view the fairness of organizational policies and choices, as well as how these views affect workers’ actions [25]. A more comprehensive understanding of fairness in an organization may also be possible through the integration of the fairness model, which incorporates distributive justice as well as procedural and interactional justice. While interactional justice stresses the fairness of interpersonal treatment and communication, procedural justice concentrates on the fairness of the procedures employed to distribute results [26].

### Significance of the study

Human capital is considered the primary corporate asset that requires good management in a competitive organizational environment. In healthcare services, where numerous systems must function in unison, good leadership practices that promote efforts to reach people’s goals in a given situation, time, and condition, transmit experiences that lead to common goals, and make people satisfied with the kind of leadership applied are essential to preventing confusion [12]. The current study’s viewpoint is crucial for deepening the understanding of paternalistic leaders who treat their employees like sons and foster the sense that they are all a part of a single, big family. There is growing awareness that these disparate management philosophies could foster emotions of nepotism in the workplace, which could compromise organizational fairness, increase staff turnover, and have an impact on care quality and performance. Thus, the purpose of this study is to look at the relationship between organizational justice and nepotism practices and paternalistic leadership styles among nurses.

### Aim of the study

#### Primary aim

- To investigate the effects of Paternalistic Leadership and Nepotism on Nurses’ Perception of Justice.

#### Secondary aim

- To assess paternalistic leadership style perception among nurses.
- To assess nepotism practices among nurses.
- To assess the organization’s justice perception among nurses.

### Research questions

- **Q1.** What is the paternalistic leadership style level among nurses?
- **Q2.** What is the nepotism practice among nurses?
- **Q3.** What is the organizational justice level among nurses?
- **Q4.** Is there a relationship between paternalistic leadership style, nepotism practice, and organizational justice?

### Theoretical framework

#### Paternalistic leadership and nepotism among nurses

Nepotism and paternalistic leadership are two important elements that affect nursing organizational dynamics. Research suggests that when applied successfully, paternalistic leadership—which combines power and kindness—can increase nurses’ loyalty and trust, which will improve their performance and job satisfaction [20, 27]. But when used improperly, it can also lead to problems like reliance and diminished autonomy. Nepotism in nursing has also been demonstrated to affect workplace equity and morale. Although some contend that nepotism may increase confidence in family ties, research indicates that it frequently erodes meritocracy, causes conflict, and has a detrimental impact on team cohesiveness [28]. Fostering an equitable and encouraging nursing work environment requires addressing these factors.

Also, it is the practice of favoring relatives or friends in the workplace, often for hiring or promotions, based on kinship rather than qualifications or merit [29]. Moreover, working under an incompetent person is a distressing situation for a nurse subject to nepotism. The inequality between the contribution rate and the benefit offered makes nurses think they work in an unfair environment. The lack of confidence that appears in such circumstances negatively affects organizational justice [30].

#### Nepotism & organizational justice

Nepotism was not considered acceptable. People’s personal beliefs that nepotism is in opposition to principles like egalitarianism, meritocracy, and self-reliance are the fundamental cause of their negative opinion of nepotism. In particular, persons with family and social ties in positions of authority are thought to benefit from nepotism. Nepotism is viewed as unjust and inappropriate in these situations when qualities other than merit or skill are prioritized [31].

According to the social exchange theory, reciprocity, trust, honesty, equality, mutual aid, and mutual benefit are the cornerstones of human relationships [32]. Working for people who are appointed to key positions irrespective of their skill level undermines the confidence that non-family members can inspire in their coworkers, reduces job satisfaction and performance, diminishes organizational efficiency, and diminishes organizational justice [33].

#### Paternalistic leadership & nepotism, and organizational justice

Coordination in healthcare settings is necessary to foster teamwork, productivity, and organizational well-being [34]. In this process, paternalistic leadership plays a significant role as a social influence strategy that encourages, energizes, and stimulates members of the organization to seek the common interest as much as possible [35]. Just treatment, fair results, and respect are all crucial paternalistic leadership traits that encourage subordinates to respond in the most advantageous way possible. Paternalistic leaders’ justice practices can have a significant impact on a range of nursing outcomes. For example, when they implement distributively, procedurally, and interactionally fair procedures, nurses’ satisfaction, positive emotions, and intrinsic motivations rise and lead to cooperative and citizenship behavior [36].

The traits of paternalistic leadership such as having a positive disposition, exercising responsibility, selflessness, discipline, and setting a good example for others. The moral principles upheld by paternalistic leaders govern the moral approach to decision-making and have the potential to establish an equitable atmosphere within an organization [37]. Paternalistic leaders improved organizational justice as a result.

On the other hand, government hospitals that are owned by the public are governed by a system known as bureaucracy [38]. Because bureaucratic culture is so pervasive, decision-making is done from the top down. The promotion of an authoritarian and paternalistic leadership style, typified by directives, nurse transfers, and a dearth of incentives and rewards, may result in a diminished sense of organizational fairness [39].

Additionally, the principles of merit, such as knowledge, skills, ability, competence, achievement, or level of education, are substituted for nepotism, kinship, friendship, or other relationships when recruiting or promoting nurses [29, 40]. Furthermore, it might be upsetting for a nurse who is a victim of nepotism to work under an incompetent person. Nurses believe they work in an unfair environment because of the disparity between the contribution rate and the benefits provided. In these situations, a lack of confidence manifests itself and has a detrimental influence on organizational justice [30].

The collectivist cultural setting in which the participants function is closely related to the acceptance of paternalistic leadership in the current study. Group cohesion, loyalty, and respect for authority are highly prized in collectivist civilizations like Egypt and many other Middle Eastern contexts. As a result, followers are encouraged to accept and even expect leaders to assume a guiding, paternal role. The ideas of paternalistic leadership, in which leaders are viewed as protective and supportive individuals who uphold authority, are consistent with this cultural perspective. As a result, nurses in collectivist cultures would view paternalistic actions as acceptable and desired leadership traits rather than as overreach, which would promote trust, compliance, and favorable views of organizational justice [41, 42].

## Method

### Design, setting, and subject of the study

For this study, a descriptive correlational research design was adopted. An analysis of the relationship or link between variables is done statistically using a descriptive correlational research approach [43]. The study was conducted in all inpatient departments at Samanoud Central Hospital, Egypt (which is affiliated with the Ministry of Health). Samanoud Central Hospital was selected as the study site due to its accessibility and convenience for data collection, as it served as the primary workplace of the researcher. This purposive sampling approach ensured feasible data gathering while maintaining the study’s focus on the target population within a major healthcare facility in the region. It consists of a main building and an outpatient clinic building with a bed capacity of 195 beds.

A convenience sample of available nurses267 was recruited for this study, consisting of those who were willing to participate and met the eligibility criteria. To ensure consistency, the inclusion criteria required nurses to have a minimum of two years of professional experience in the field, regardless of age, gender, or educational background. This threshold was set to ensure that participants possessed sufficient practical experience to provide meaningful insights relevant to the study.

However, the limited number of male nurses in the sample reflects the broader context in Egypt, where nursing has traditionally been a female-dominated profession and male participation has only recently increased, leading to their underrepresentation in hospital settings and consequently in this study.

Practical concerns led to the choice of a convenience sampling technique. By using this strategy, the researchers were able to effectively find volunteers who were available and within the study’s budget and time limits. Convenience sampling is practical and speeds up data gathering, but it has drawbacks, such as the possibility of selection bias. Because of this bias, the results may not accurately represent the larger nursing community, particularly with regard to gender diversity and professional roles, which could compromise the sample’s representativeness.

#### Data collection

Three tools were used for data collection.

#### Instrument one

The Paternalistic Leadership Questionnaire consists of two parts.

#### Part (1)

This part includes personal characteristics of staff nurses such as age, gender, educational qualification, and years of experience.

**Part (2**) It was developed by Cheng et al. (2004) [44]. aims to measure paternalistic leadership style in the work environment. It consisted of 15 items measuring paternalistic leadership. it represented in three dimensions of paternalistic leadership as follows: Benevolent leadership (5 items), Moral leadership (5 items), and Authoritarian leadership (5 items). Their responses were measured by using a five-point Likert scale ranging from 1 (strongly disagree) to 5(strongly agree).

**Paternalistic Leadership Questionnaire scoring system** Total score of paternalistic leadership style was divided into three levels based on a cut point of % 60% as follows: Low-level value < 60%, Moderate level 60%-75% and High level > 75% [45].

### Instrument two: Nepotism Questionnaire

This questionnaire was constructed by Arasli, Bavik, & Ekiz, (2006) [46]. and aims to measure nepotism practices in the workplace. The total items is 12; their responses were measured by using a five-point Likert scale ranging from 1 (strongly disagree) to 5(strongly agree).

**Nepotism Questionnaire scoring system** The total score of nepotism practices was divided into three levels based on a cut point of % 60 as follows: Low-level value < 60%, Moderate level 60%-75%, and High level > 75% [47].

### Instrument three: Organizational Justice Questionnaire

This questionnaire was developed by Karriker &Williams’ (2009) [48]. aims to measure organizational justice in the workplace. It consisted of 44 items measuring organization justice it representing five dimensions of organization justice as follows: Agent-Referenced Distributive Justice (7 items), Agent-Referenced Procedural Justice (8 items), Agent-Referenced Interpersonal Justice (11 items), System-Referenced Distributive Justice (7 items) and System-Referenced Procedural Justice (11 items). Their responses were measured by a five-point Likert scale ranging from 1 (strongly disagree) to 5(strongly agree).

**Organizational justice questionnaire scoring system** The total score of organizational justice was divided into three levels based on a cut point of % 60% as follows: Low-level value < 60%, Moderate level 60%-75% and High level > 75% [49].

### Translation procedures and the tool’s validity and reliability

The three instruments of the Paternalistic Leadership Questionnaire, Nepotism Questionnaire, and Organizational Justice Questionnaire were translated into Arabic using the process of translation and back-translation Brislin, (1970) [50]. The translated tools were reviewed by a panel of seven professors/assistant professors from the university’s nursing academic staff. Furthermore, the panel reviewed the tools for clarity, relevancy, applicability, comprehensiveness, understanding, and ease of implementation after they were translated into Arabic and content and face validity. Modifications were then implemented based on their recommendations.

Reliability was estimated among 26 nurses using the test-retest method two weeks apart. Then Cronbach’s alpha reliability test was done using the SPSS software. Cronbach’s alpha coefficients were calculated for multipoint items to evaluate the measurement reliability. Using Cronbach’s alpha, which requires a value of at least 0.5 and ideally more than 0.7. Cronbach’s alpha in its original version was for each instrument was as follows: (α = 0. 897) for Internal consistency of the first instrument *(paternalistic Leadership Questionnaire)*, (α = 0.865) for Internal consistency of the second instrument *(Nepotism Questionnaire)*, and (α = 0.930) for Internal consistency of the third instrument *(Organizational Justice Questionnaire*). The results of the Cronbach alpha reliability test for the three instruments indicated that they were reliable in detecting the objectives of the study.

### Pilot study

Twenty-five nursing staff members, or 10% of the study population, participated in a pilot study. The study aimed to assess the validity and comprehensibility of the statement in addition to the amount of time needed to finish the surveys. None of the nurses who participated in the pilot experiment was part of the main study sample. Two of the most significant modifications made in response to the pilot research are rewording and clarity to comply with Egyptian culture.

### Data collection

The process of gathering data spanned three months, starting in December 2023 and ending in February 2024. Each study participant received a questionnaire from the researcher during their morning and afternoon shifts at work, which allowed for the collection of data. The researcher described the study’s purpose and the filling instrument. The questionnaire sheet has to be filled out in 20 to 30 min. Every day, between ten and fifteen questionnaires from nursing staff were collected. Three days a week, the researcher visited the hospital. To ensure that all of the researcher’s inquiries were addressed, nursing personnel completed the questionnaire sheets.

### Ethics approval and consent to participate in the manuscript

This study was conducted following the ethical principles outlined in the Declaration of Helsinki (DoH Oct 2008). The Mansoura University Faculty of Nursing’s Research Ethics Committee, Egypt, granted ethical permission. (No. 364 − 23). All necessary information about the study was introduced in the first section of the sheet. The questionnaire included a statement related to the aim and nature of the study. All participants gave verbal agreement and filled in the pen and paper-administered questionnaire. the questionnaire involved asking the participants to agree to give their informed consent before beginning their response to the sheet. The respondents were guaranteed the privacy and confidentiality of their answers, the voluntary nature of their involvement, and the fact that their absence would not hurt their grades or result in any negative outcomes. This ensures that participants can freely choose to participate without the fear of academic repercussions. It was determined that participants had the right to withdraw from the study at any time. The right to withdraw was communicated both verbally and in writing at the outset of the study.

### Statistical approach

Using SPSS software (Statistical Package for the Social Sciences, version 22, SPSS Inc., Chicago, IL, USA), the gathered data were arranged, tabulated, and statistically examined. The normality assumption was tested and accepted. Normality was assessed using both graphical methods and statistical tests. The results indicated that the data did not significantly deviate from a normal distribution. An international measure of reliability, Cronbach’s Alpha test, was used to evaluate reliability statistics. Frequency and percentage were used to express the category variables. The mean and standard deviation were used to represent Continuous variables. The independent t-test was employed to examine the disparity between two continuous variable means. To compare the differences between more than two continuous variable means, the ANOVA test was employed. A test of the Pearson correlation coefficient was used to determine whether two continuous variables were related. For data visualization, graphs were created with SPSS and Microsoft Excel. A p-value of 0.05 and 0.01 was designated as the significant level.

## Results

Table 1 demonstrated the studied nurses’ characteristics. According to the table, nearly two-thirds of the studied nurses were (61%) aged between 25 and 35 years, whereas 20.3% were aged 18–34 years and the others (18.7%) were aged more than 35 years. Regarding gender, more than three-quarters of the studied nurses were female (76.4%). Moreover, more than one-third of the nurses in the study (36.4%) with bachelor’s degrees in nursing science. While (39.3%) had 3–5 years of experience.

**Table 1:** Personal characteristics of the studied nurses (*n* = 267)

Variables	No.	%
Age in Years		
18: 24 years	54	20.3
25:35 years	163	**61.0**
> 35 years	50	18.7
Gender		
Male	63	23.6
Female	204	**76.4**
Level of education		
School Nursing	4	1.5
Technical institute of nursing	164	61.4
Bachelor degree	97	**36.4**
Other	2	0.7
Years of experience		
> 2 year	30	11.2
3:5 year	105	**39.3**
6:10 years	73	27.3
> 10 years	59	22.2

Table 2 illustrates the variations in mean scores of paternalistic leadership and nepotism practices in relation to organizational justice across the personal characteristics of the nurses under study. The results indicated a highly statistically significant relationship between age and gender with paternalistic leadership, as well as a significant correlation between gender and nepotism practices. Organizational justice was also found to be significantly associated with nurses’ age, gender, and educational level.

Age had a significant effect on paternalistic leadership (F = 6.084, *p* = 0.003, η² = 0.05), indicating a medium effect size. Additionally, female nurses reported higher perceptions of organizational justice compared to males (t = 5.268, *p* < 0.001, d = 0.64), reflecting a medium-to-large effect size.

**Table 2:** Mean score differences of paternalistic leadership, nepotism practice, and organizational justice with personal characteristics of the studied nurses (*n* = 267) – with effect sizes

Variables	Paternalistic leadership (Mean ± SD)	Nepotism practice (Mean ± SD)	Organizational justice (Mean ± SD)	Effect size
Age in Years				
18–24 years	48.46 ± 9.97	43.59 ± 8.92	156.91 ± 22.91	
25–35 years	46.34 ± 9.25	42.18 ± 8.82	148.07 ± 27.33	
> 35 years	51.16 ± 4.59	43.62 ± 8.30	162.82 ± 30.41	
F / p-value	6.084 / 0.003**	**0.846 / 0.430**	6.502 / 0.002**	**η² = 0.05 (medium)**
Gender				
Male	45.49 ± 9.99	39.19 ± 9.92	137.32 ± 30.24	
Female	48.35 ± 8.46	43.83 ± 8.06	157.35 ± 25.08	
t / p-value	2.241 / 0.026*	3.771 / <0.001**	5.268 / <0.001**	**d = 0.28 (small)**,** d = 0.46 (medium)**,** d = 0.64 (medium-large)**
Education level				
School Nursing	50.0 ± 2.94	49.5 ± 3.7	179.25 ± 5.25	
Technical degree	47.7 ± 8.93	43.17 ± 8.69	155.85 ± 25.26	
Bachelor degree	47.51 ± 9.15	41.77 ± 8.82	146.45 ± 30.71	
Other	49.5 ± 3.54	40.0 ± 14.14	133.5 ± 12.02	
F / p-value	0.130 / 0.942	1.397 / 0.244	4.038 / 0.008**	η² = 0.04 (small–medium)
Years of Experience				
> 2 years	46.13 ± 10.99	46.43 ± 10.03	158.63 ± 24.21	
3–5 years	48.3 ± 9.33	42.43 ± 7.52	150.34 ± 26.69	
6–10 years	46.14 ± 8.18	41.86 ± 9.37	149.07 ± 28.38	
> 10 years	49.24 ± 7.54	42.47 ± 9.04	158.02 ± 29.47	
F / p-value	1.822 / 0.144	2.118 / 0.098	1.876 / 0.134	

Table 3 demonstrates the correlation between paternalistic leadership style, nepotism practices, and organizational justice. The findings revealed a statistically significant positive correlation between overall paternalistic leadership style and organizational justice (*r* = 0.549, *p* < 0.001), indicating a large effect size. In contrast, a statistically significant negative correlation was observed between overall nepotism practices and organizational justice (*r* = − 0.337, *p* < 0.001), suggesting that higher levels of nepotism are associated with lower perceptions of organizational justice.

**Table 3:** Correlation between paternalistic leadership style, nepotism practice with organizational justice among the studied nurses (*n* = 267)

	Organizational justice	
	*r*	*P*
Paternalistic Leadership Style	0.549	(< 0.001) **
Nepotism practice	-0.337	(< 0.001) **

**Correlation is significant at the 0.05 level (2-tailed)

Table 4 demonstrates significant correlations among paternalistic leadership styles, nepotism practices, and organizational justice dimensions. Benevolent and moral leadership showed strong positive associations with total paternalistic leadership (*r* = 0.833 and *r* = 0.841, *p* < 0.001), highlighting their central role in shaping this leadership construct. In contrast, authoritarian leadership was negatively correlated with most justice dimensions, particularly procedural (*r*=-0.218, *p* < 0.001) and total organizational justice (*r*=-0.212, *p* < 0.001), suggesting its detrimental impact on fairness perceptions. Nepotism practice revealed moderate positive correlations with distributive (*r* = 0.560, *p* < 0.001), procedural (*r* = 0.551, *p* < 0.001), and interpersonal justice (*r* = 0.464, *p* < 0.001), indicating that while nepotism may reinforce some relational aspects, it simultaneously undermines impartial justice. Importantly, the strongest correlations were observed between organizational justice dimensions themselves, with total organizational justice strongly linked to procedural (*r* = 0.862, *p* < 0.001) and interpersonal justice (*r* = 0.858, *p* < 0.001), underscoring the interdependence of fairness perceptions across multiple domains.

**Table 4:** Correlation matrix between paternalistic leadership style, nepotism practice, and organizational justice (*n* = 267)

Item		B L leadership	M.L	AL	TPL	NP	DJ	PJ	IJ	SDJ	SPJ	TOJ
Benevolent leadership	r	1										
	p											
Moral leadership (M.L)	r	0.784	1									
	p	(< 0.0001)**										
Authoritarian leadership	r	-0.106	-0.069	1								
	p	0.084	0.260									
Total paternalistic leadership	r	0.833	0.841	0.378	1							
	p	(< 0.0001)**	(< 0.0001)**	(< 0.0001)**								
Nepotism practice	r	0.247	0.354	-0.156	0.221	1						
	p	(< 0.0001)**	(< 0.0001)**	0.011*	(< 0.0001)**							
Distributive Justice	r	0.358	0.384	-0.117	0.310	0.560	1					
	p	(< 0.0001)**	(< 0.0001)**	0.056	(< 0.0001)**	(< 0.0001)**						
Procedural Justice	r	0.395	0.453	-0.218	0.315	0.551	0.654	1				
	p	(< 0.0001)**	(< 0.0001)**	(< 0.0001)**	(< 0.0001)**	(< 0.0001)**	(< 0.0001)**					
Interpersonal Justice	r	0.352	0.421	-0.166	0.302	0.464	0.587	0.744	1			
	p	(< 0.0001)**	(< 0.0001)**	0.006**	(< 0.0001)**	(< 0.0001)**	(< 0.0001)**	(< 0.0001)**				
System Distributive Justice	r	0.289	0.326	-0.191	0.213	0.498	0.722	0.640	0.579	1		
	p	(< 0.0001)**	(< 0.0001)**	0.002**	(< 0.0001)**	(< 0.0001)**	(< 0.0001)**	(< 0.0001)**	(< 0.0001)**			
System Procedural Justice	r	0.356	0.435	-0.211	0.290	0.519	0.650	0.668	0.639	0.752	1	
	p	(< 0.0001)**	(< 0.0001)**	0.001**	(< 0.0001)**	(< 0.0001)**	(< 0.0001)**	(< 0.0001)**	(< 0.0001)**	(< 0.0001)**		
Total organizational Justice	r	0.411	0.477	-0.212	0.337	0.600	0.824	0.862	0.858	0.845	0.877	1
	p	(< 0.0001)**	(< 0.0001)**	(< 0.0001)**	(< 0.0001)**	(< 0.0001)**	(< 0.0001)**	(< 0.0001)**	(< 0.0001)**	(< 0.0001)**	(< 0.0001)**	

*Correlation is significant at the 0.01 level (2-tailed)

**Correlation is significant at the 0.05 level (2-tailed)

**Fig. 1:**
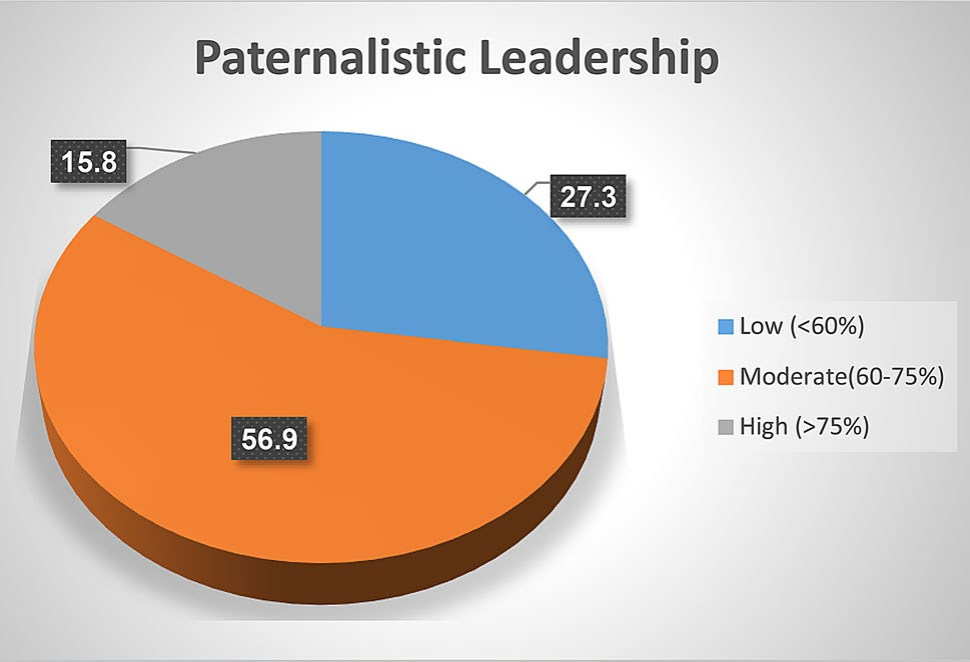
Levels of paternalistic leadership as perceived by studied nurses (*n* = 267)

Fig. 1: showed levels of paternalistic leadership as perceived by studied nurses. The figure revealed that more than half (56.9%) of studied nurses perceived a moderate level of paternalistic leadership, whereas 15.8% of studied nurses had a low level.

**Fig. 2:**
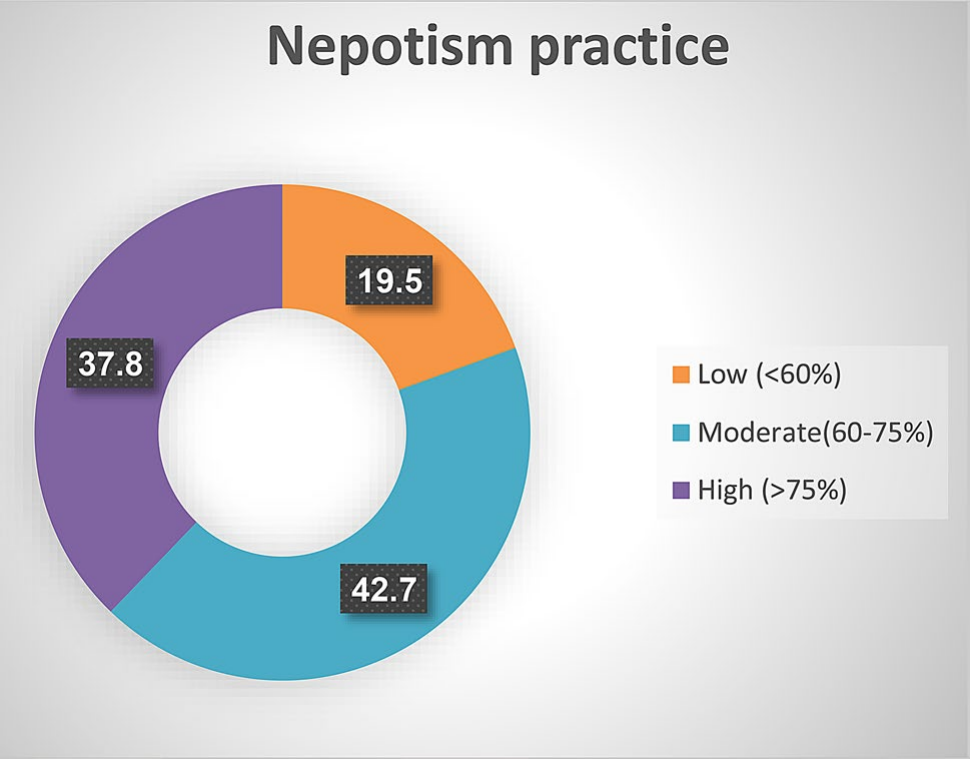
Levels of nepotism practice as perceived by studied nurses (*n* = 267)

Fig. 2 displayed levels of nepotism practice as perceived by studied nurses. The figure displayed that 42.7% of studied nurses perceive a moderate level of nepotism practice, whereas 19.5% had a low level by studied nurses.

**Fig. 3:**
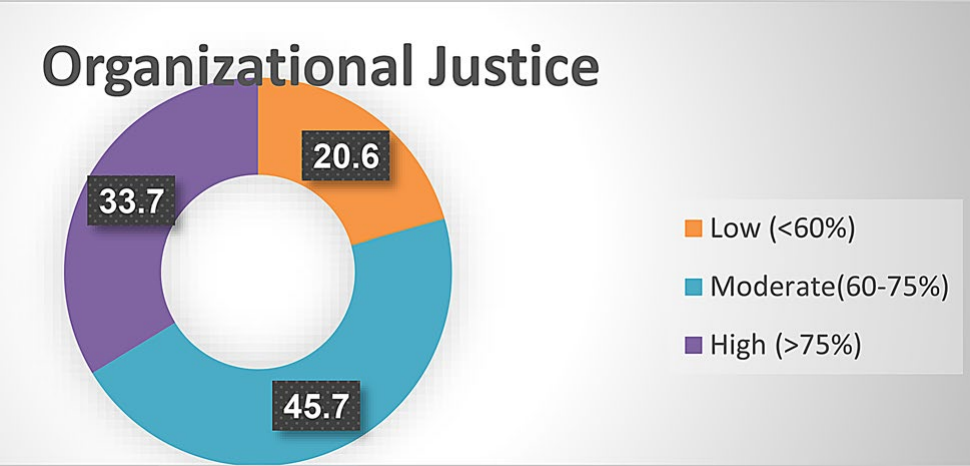
Levels of organizational justice as perceived by studied nurses (*n* = 267)

Fig. 3: showed levels of organizational justice as perceived by studied nurses. The figure displayed that 45.7% of studied nurses perceived a moderate level of organizational justice whereas 20.6% had a low level of studied nurses.

**Fig. 4:**
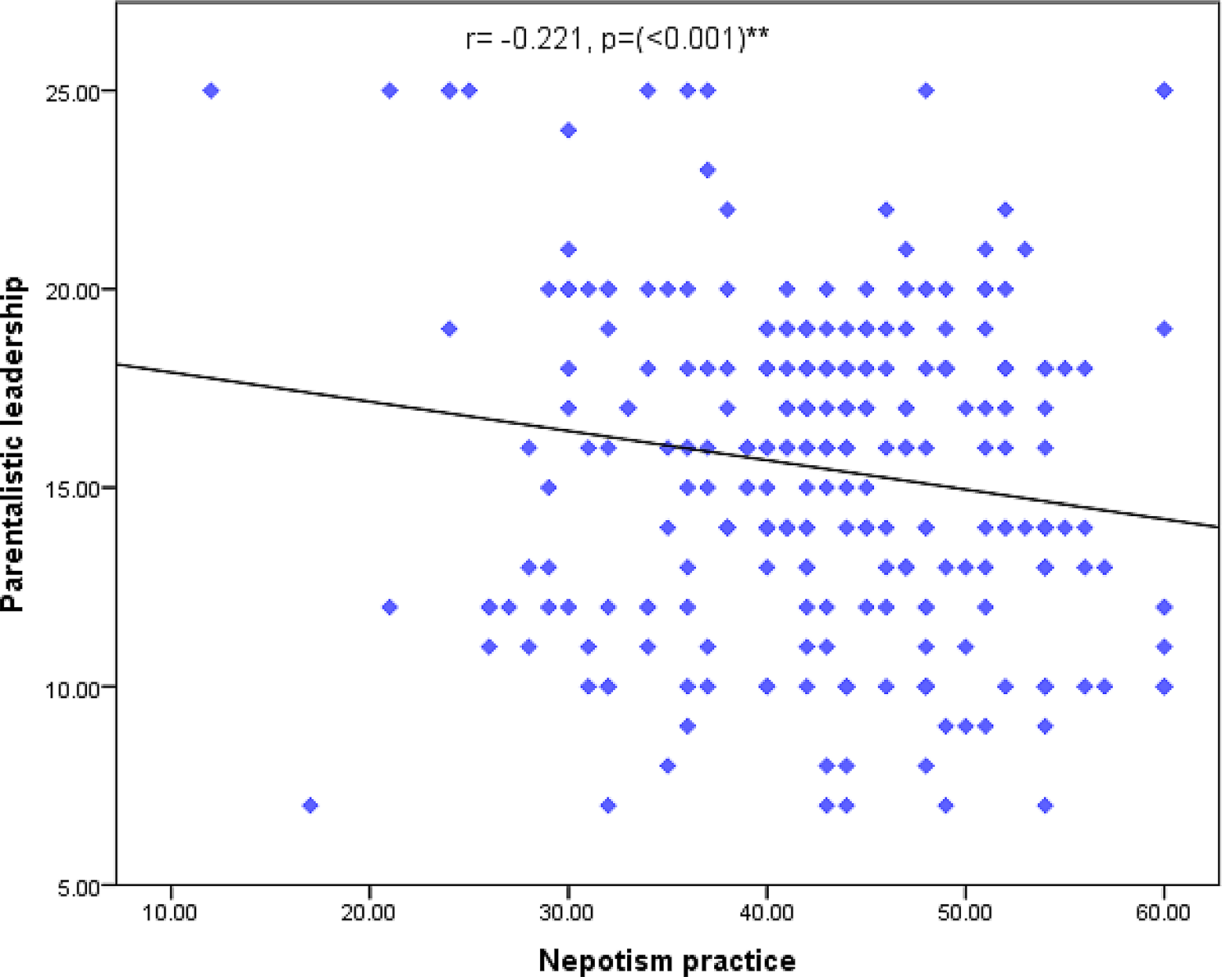
Correlation between total score of paternalistic leadership style and nepotism practice of the studied nurses

Fig. 4 displays correlation between total paternalistic leadership style and nepotism practice. It can be noticed from the figure that there was a highly statistical significant negative relationship between total score of paternalistic leadership style and nepotism practice of the studied nurses.

**Fig. 5:**
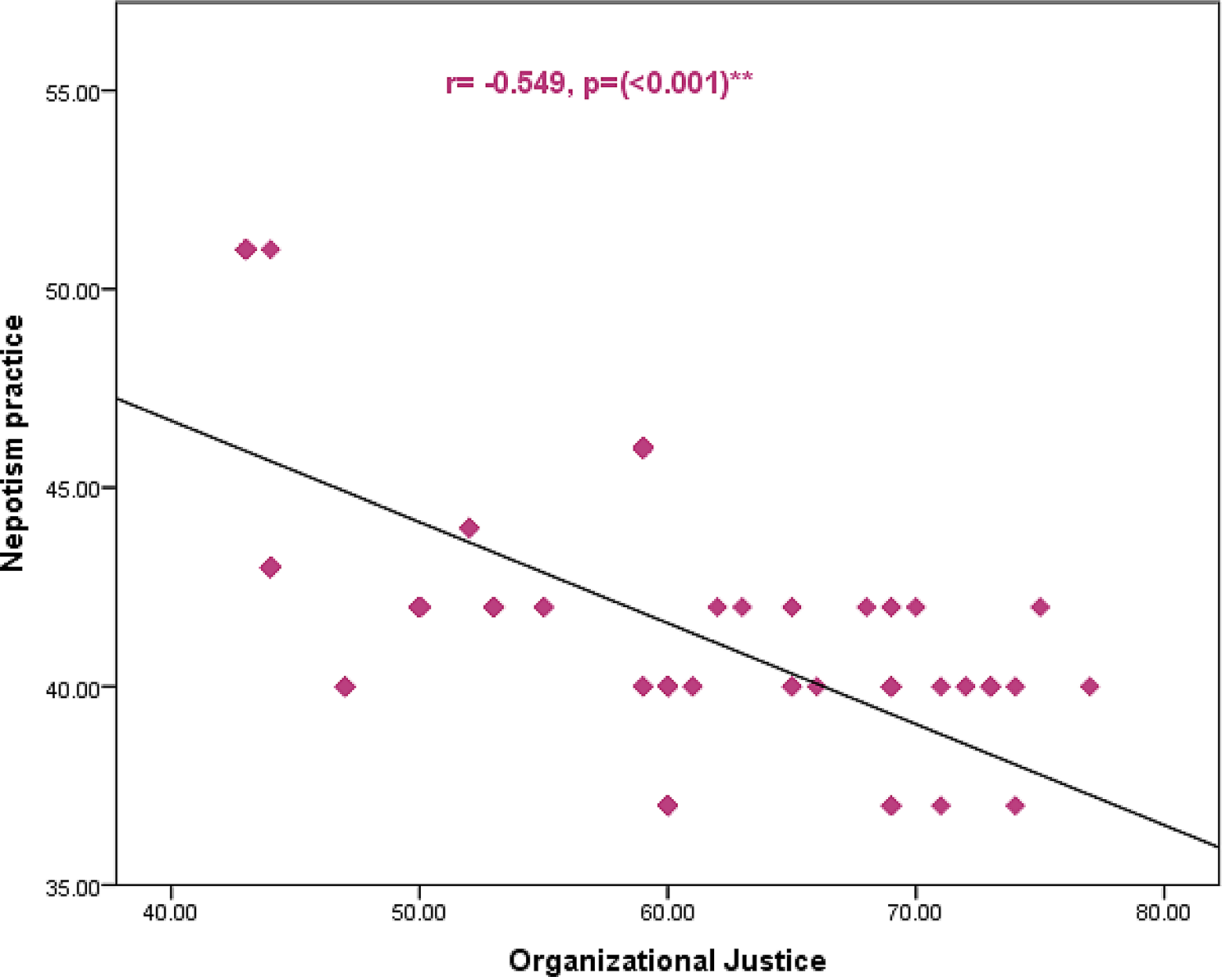
Correlation between total organization justice and nepotism practice of the studied nurses

Fig. 5 displays correlation between total organization justice and nepotism practice of the studied nurses. The figure displayed that there was a highly statistical significant negative relationship between total score of organization justice and nepotism practice of the studied nurses.

**Fig. 6:**
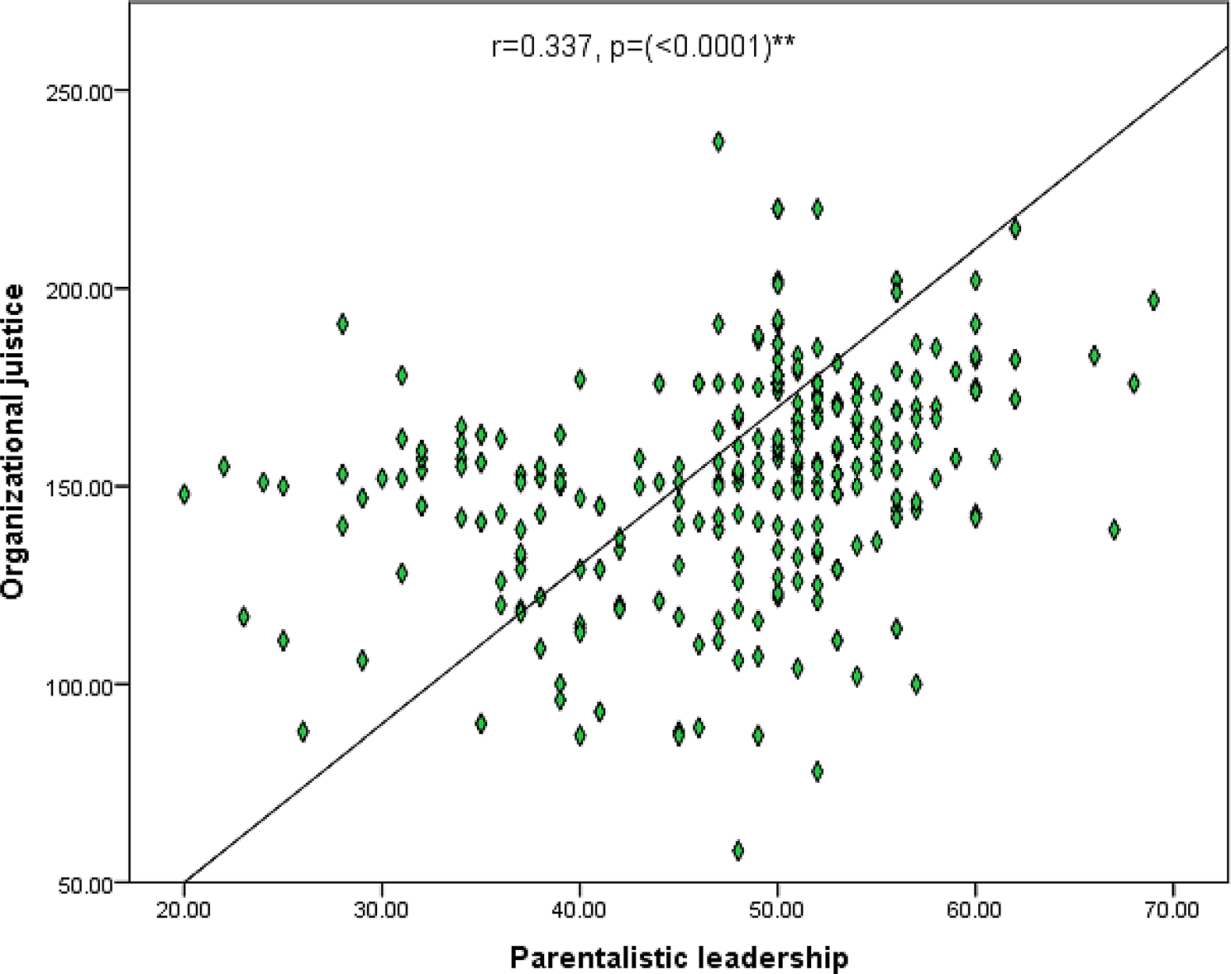
Correlation between total paternalistic leadership style and total organizational justice of the studied nurses (*n* = 267)

Fig. 6 illustrates the correlation total paternalistic leadership style and the total organizational justice of the studied nurses. It can be observed from the figure that there was a highly statistically significant positive relationship between the total score of paternalistic leadership style and total organizational justice of the studied nurses.

## Discussion

The metaphor of a father and son is commonly used in literature to create a family atmosphere within a corporation and to communicate closely with employees. Additionally, workers must have an accurate impression of the workplace in terms of harmony between employers and employees, as well as loyalty to the organization [51]. Because most family firms lack an institutional structure that could affect organizational justice, the concept of nepotism can have a substantial impact on employees. In light of this, the present study investigates paternalistic leadership style, nepotism practice, and its relation to organizational justice among nurses. The findings of this study will be discussed as follows:

**Regarding paternalistic leadership**, the present study discovered that more than half of the studied nurses perceive moderate levels of paternalistic leadership. This might be explained by the fact that some This may be because some employees reported that their managers provide help during emergencies, show care for long-serving staff, and demonstrate moral behavior. But some of them showed that their supervisors determine all choices in the organization, whether they are important or not. In the same line, the result of [52] concluded that administrators behave like the senior of a large family, treat employees like sons and daughters, and expect them to get al.ong with one another like brothers and sisters in an attempt to foster a family atmosphere at work. This led to a moderate level of general employee perceptions about paternalistic leadership. These results were in disagreement with [53], who discovered that health worker perceptions of paternalistic leadership were low, as the employees stated a moderate level of employee performance perceptions. This difference may be due to contextual variations in leadership culture across countries or healthcare systems.

### Regarding the nepotism practice in the workplace

Also, the present study exposed that less than half of the studied nurses perceive a moderate level of nepotism practice. According to a study by nurses, the hospital provides appropriate and formal training to employees to help them get ready for their professions, use the outcomes of the educational programs to meet goals, and base the development of services on these outcomes. Some of the nurses studied showed that during the employment process, the hospital doesn’t clarify both the positive and the negative aspects of the job. On the same line with the result of [54], who found that nepotism perception had a moderate level and described that this was an important level, even though not very high. Also, these results were in congruence with [55], who found that perceived nepotism had a moderate level among studied employees and stated that lower levels of organizational trust are caused by higher perceived levels of nepotism. This result is contradicted by [56], who stated that there were high levels of nepotism in hospitality. The findings demonstrated that, for the majority of workers, nepotism originates from a lack of equity and clarity in the organization’s rules, policies, and procedures.

### Regarding organizational justice

Also, the present study discovered that less than half of the studied nurses perceive a moderate level of organizational justice. This is not surprising, as Egyptian nurses frequently deal with difficult working conditions and minimal institutional support. In addition to other difficulties, including personnel shortages, heavy workloads, and inadequate equipment, low pay deters nurses from feeling motivated. Additionally, nursing management has not treated them fairly, did not participate in decision-making, and nurses are not satisfied with their payment and incentives. The result is matched with [57], who stated that the common nurses in this study were found to perceive moderate levels of organizational justice since it was shown that most nurses in this study were unhappy with their jobs because they received little assistance.

On the same line with the result of [58], who found that nurses had moderate levels of organizational justice as nurses were more comfortable with their jobs at their healthcare organization. which is in congruence with [59], who also specified that nurses had moderate levels of organizational justice and described that Fair staff treatment and a workplace environment provide the opportunity for personal growth of self-confidence and coping abilities within the workplace. Also, this result aligns with the results of [60], who reported that nurses’ perceptions of organizational justice were below average and emphasized that healthcare organizations should address these perceived injustices to improve nurse satisfaction and minimize workplace deviance.

### The results of the study regarding the relationship between paternalistic leadership style dimensions and nepotism practice

The finding of the study showed that there was a highly statistically significant negative correlation between benevolent leadership dimension, moral leadership dimension & authoritarian leadership dimension and nepotism practice of the studied nurses. This suggests that leadership styles focusing on fairness, ethics, and control may reduce the occurrence of nepotism. Benevolent and moral leaders, by prioritizing employee welfare and ethical decision-making, discourage favoritism. Similarly, authoritarian leaders, while focused on maintaining control, are less likely to engage in nepotism, possibly because they prioritize organizational norms over personal relationships. These leadership styles contribute to a more equitable work environment, minimizing nepotistic practices.

On the same line with the result of [11] who said that it was discovered that nepotism in post-hiring procedures was also negatively correlated with paternalistic leadership traits. outlined how employees don’t perceive any discriminatory tactics or nepotism in the post-hiring processes when they believe their supervisor cares about their well-being and addresses their performance issues. Also, this result was harmonized with the result of a study performed by [22], who discovered that paternalistic leadership has a negative relationship with nepotism. This finding showed that nepotism is significantly and negatively impacted by authoritarian leadership in recruitment, significantly and negatively impacted by benevolent leadership in promotion, and negatively impacted by moral leadership in relationships. Thus, moral leadership lessens the perception of nepotism in interpersonal connections.

### The result of the study regarding the relationship between organizational justice and nepotism practice

The study revealed a highly statistically significant negative correlation between all organizational justice dimensions (Distributive Justice, Procedural Justice, Interpersonal Justice, System Distributive Justice & System Procedural Justice) and the nepotism practice of the studied nurses. This correlation can be rationalized by understanding that perceptions of organizational justice may create a work environment where employees feel compelled to reciprocate fairness with loyalty to specific individuals or groups, potentially fostering nepotistic behavior. Additionally, in organizations where systemic distributive and procedural justice is emphasized, employees may interpret these as mechanisms that inadvertently permit favoritism, especially if formal policies are inconsistently enforced or lack transparency.

Research supports the interplay between organizational justice and workplace behaviors, including nepotism. For instance [16], highlighted that organizational justice influences both positive and negative workplace outcomes, depending on the context and the degree to which fairness principles are applied universally. Similarly [61], discussed how interpersonal and procedural justice might shape employees’ attitudes, sometimes leading to unintentional favoritism under the guise of maintaining relationships or fulfilling social obligations. These findings stress the importance of ensuring not just the perception but also the practice of justice in organizations to mitigate behaviors like nepotism, which can erode trust, equity, and performance in healthcare settings.

This result was coordinated with the result of a study performed by [62] who noted that the main perception of nepotism was procedurally unfair, with distributive unfairness resulting from the unfair process. The study’s findings show that an organization’s perception of nepotism can harm organizational justice. He discovered a correlation between perceived nepotism and a worse opinion of the organization’s culture, a decline in trust, and a greater sense of secrecy within the organization. On the same line with the result of [63], who proposed that favoritism, or nepotism, negatively impacts procedural justice, distributive justice, interactional justice, and job embeddedness.

### The results of the study regarding nursing staff perception of paternalistic leadership, nepotism practice, and organizational justice in relation to their personal characteristics

The findings of this study highlight significant associations between nurses’ sociodemographic characteristics and their perceptions of paternalistic leadership, nepotism, and organizational justice. These results contribute to understanding how individual differences influence workplace perceptions and dynamics in healthcare settings.

Related to paternalistic Leadership, the study revealed a highly statistically significant relationship between age and gender with perceptions of paternalistic leadership. Younger nurses and male nurses may perceive paternalistic leadership differently, potentially due to generational differences in leadership expectations or gender-based social dynamics. The same is in line with the result of [64] who definite that there was a significant relation between paternalistic leadership and gender. Additionally, these results are consistent with research like [65], which found that while older employees may see paternalistic leadership as protective and supportive, younger employees tend to view it negatively, seeing it as too controlling. Similarly, research by [66] has demonstrated gendered perceptions of paternalistic leadership, finding that women seem to value the caring components of this style more than males. Our findings, however, contrast with those of studies such as [67] which proposed that cultural values, rather than personal traits like age or gender, are the main factor influencing perceptions of paternalistic leadership. Differences in organizational and cultural environment could be the cause of this disparity, which calls for more research.

As regarded Nepotism practice, A significant correlation was observed between gender and perceptions of nepotism. Female nurses in our study may have reported higher sensitivity to nepotistic practices, potentially due to historical biases in professional advancement opportunities for women in male-dominated hierarchies. These outcomes are consistent with research by [68] which showed that women are frequently more conscious of and adversely impacted by workplace favoritism. On the other hand, our findings are different from those of [69] who discovered no discernible gender differences in how employees view nepotism, indicating that favoritism affects all workers equally. The gender distribution of the workforce in the contexts under study and different corporate cultures may be the cause of this discrepancy. Additionally, there was no statistically significant correlation between the study participants’ personal traits and their nepotism practices, with the exception of gender, which was very significant. On the same line with the result of [70] who discovered that, on average, women outperformed males in terms of transactional nepotism, promotion nepotism, and recruiting process nepotism. also, the study exposed that organizational justice was significantly correlated with the studied nurses’ age, gender, and educational level.

In relation to Organizational Justice, the study also found that age, gender, and educational level were significantly associated with perceptions of organizational justice. Older nurses and those with higher educational attainment likely expect greater fairness and transparency in decision-making and resource allocation. These results are in line with a study by [71] that showed how demographic characteristics affect how distributive, procedural, and interactional justice are seen. On the same in line with the result of [72] who claimed that the relationship between justice aspects and deviance is moderated by employee age.

## Conclusion

Based on the study’s findings, the organizational justice perceived by nurses were found to have a highly statistically significant positive relationship with paternalistic leadership dimensions. Additionally, this result revealed a highly statistically significant negative correlation between all organizational justice dimensions and the nepotism practices of the examined nurses. Finally, Future research should look at how different leadership philosophies, like authentic, ethical, and responsible leadership, affect the connection between OJ and nepotism among nurses. To investigate why there are so differing opinions about the link between the variables in this study, a qualitative technique might also be used.

### Study limitations

This study encountered two main limitations: The use of convenience sampling may affect the generalizability of the findings, as the selected sample may not accurately represent the wider population of nurses across different healthcare settings. The cross-sectional design limits the ability to establish causal relationships between paternalistic leadership, nepotism, and organizational justice. Future longitudinal studies are recommended to provide stronger evidence of causality.

### Recommendations

Based on the findings of this study, several recommendations are proposed to improve leadership practices, reduce nepotism, and strengthen organizational justice within nursing settings. Nursing managers are encouraged to maintain continuous communication with staff nurses, carefully listening to their needs, opinions, and concerns in relation to paternalistic leadership practices. Such open dialogue fosters a supportive and inclusive work environment.

To decrease nepotism practices, it is essential to ensure transparency and clarity in decision-making processes related to selection, appointment, and promotion policies. Clearly defining tasks and responsibilities within job descriptions, maintaining a fair distribution of workloads, and applying the principle of merit in performance evaluation and promotions are crucial steps to enhance fairness and motivation among nurses. Additionally, building relationships based on trust, respect, and teamwork contributes to a healthier organizational climate. Establishing an effective system for evaluating nursing performance and utilizing these evaluations in administrative activities is also recommended. Leaders should be trained to recognize and avoid nepotism, while bias mitigation workshops can be introduced to combat unconscious bias in managerial decisions.

Enhancing awareness of organizational justice among nursing staff can be achieved by developing fair incentive systems linked to clear performance indicators. At the same time, nursing management should promote integrity and transparency in communication while addressing systemic barriers to justice. Equitable access to promotions and rewards must be ensured, alongside consolidating the principles of equality, fairness, and justice across all managerial decisions. Furthermore, motivational programs grounded in ethical behaviors, as well as effective control systems to monitor and reduce nepotism, should be implemented. Regular reports on deviations from ethical standards can be submitted to managers and staff, while supportive structures for work ethics should be strengthened.

Another recommendation is to develop a comprehensive onboarding program for newly hired nurses, which should include orientation sessions, job-specific training, and mentorship opportunities. These components will familiarize staff with paternalistic leadership practices, strategies to prevent nepotism, and principles of organizational justice. Encouraging moral behavior among both leaders and staff, avoiding favoritism, and ensuring fairness in interpersonal and professional interactions will contribute to a more positive work environment, which in turn enhances staff satisfaction and work quality.

Finally, the study also highlights methodological considerations. The reliance on self-reported questionnaires may have introduced social desirability bias, as participants might have provided responses perceived as socially acceptable rather than fully accurate. Moreover, the use of a cross-sectional design restricts the ability to establish causal relationships between paternalistic leadership, nepotism, and organizational justice. Future research is therefore recommended to adopt longitudinal designs to better understand these complex associations over time.

## Data Availability

The data that support the findings of this study are available from the corresponding author upon reasonable request. Also, all data generated or analyzed during this research are included in this manuscript.
